# Diet Can Impact Microbiota Composition in Children With Autism Spectrum Disorder

**DOI:** 10.3389/fnins.2018.00515

**Published:** 2018-07-31

**Authors:** Kirsten Berding, Sharon M. Donovan

**Affiliations:** ^1^Division of Nutritional Sciences, University of Illinois, Urbana, IL, United States; ^2^Department of Food Science and Human Nutrition, University of Illinois, Urbana, IL, United States; ^3^Carl R. Woese Institute for Genomic Biology, University of Illinois, Urbana, IL, United States

**Keywords:** autism, microbiota, dietary patterns, nutrients, feeding behavior

## Abstract

Diet is one of the most influential environmental factors in determining the composition of the gastrointestinal microbiota. Microbial dysbiosis in children with Autism Spectrum Disorder (ASD) and the impact of some bacterial taxa on symptoms of ASD has been recognized. Children with ASD are often described as picky eaters with low intake of fiber-rich foods, including fruits and vegetables. However, the impact of diet on the microbiota composition in children with ASD is largely unknown. Herein, fecal samples, 3 day food diaries and the Youth and Adolescence Food Frequency questionnaire (YAQ) were collected from children with ASD (ASD; *n* = 26) and unaffected controls (CONT; *n* = 32). Children's ASD symptoms were determined using the Pervasive Developmental Disorder Behavior Inventory Screening Version (PDDBI-SV). Differences in the microbiota composition at the phyla, order, family, and genus level between ASD and CONT were observed. Microbiota composition of children with ASD was investigated in relation to feeding behavior, nutrient and food group intake as well as dietary patterns derived from the YAQ. In children with ASD, two distinct dietary patterns (DP) were associated with unique microbial profiles. DP1, characterized by higher intakes of vegetables, legumes, nuts and seeds, fruit, refined carbohydrates, and starchy vegetables, but lower intakes of sweets, was associated with lower abundance of *Enterobacteriaceae, Lactococcus, Roseburia, Leuconostoc*, and *Ruminococcus*. DP2, characterized by low intakes of vegetables, legumes, nuts and seeds and starchy vegetables, was associated with higher *Barnesiellaceae* and *Alistipes* and lower *Streptophyta*, as well as higher levels of propionate, isobutyrate, valerate, and isovalerate. Peptostreptococcaceae and *Faecalibacterium* predicted social deficit scores in children with ASD as measured by the PDDBI-SV. Diet-associated microbial profiles were related to GI symptoms, but no significant interaction between nutrition and microbiota in predicting social deficit scores were observed. In conclusion, dietary patterns associated with fecal microbiota composition and VFA concentrations in children with ASD were identified. Future studies using a larger sample size and measuring other behaviors associated with ASD are needed to investigate whether dietary intake may be a modifiable moderator of ASD symptoms.

## Introduction

The gastrointestinal (GI) microbiota is influenced by a variety of environmental factors, including geographical region, presence of pets in the household and dietary factors. It has been estimated that more than 50% of microbial changes can be attributed to diet (Zhang et al., 2010). Short-term changes of dietary intake over a 5 day period have shown to change the composition and function of the human GI microbiota, while habitual dietary patterns are thought to be more notable associated with long-term stability of the GI microbiota (Wu et al., 2011; David et al., 2014). We have previously demonstrated that habitual dietary patterns are associated with a distinct microbial profile and microbial stability over a 6 months period in 4–8 year-old children (Berding et al., 2018).

In recent years, the GI microbiota has been implicated as a potential pathway affecting symptom manifestation in cognitive and neurodevelopmental disorders, such as anxiety, depression and Autism Spectrum Disorder (ASD). Aberrations in the GI microbiota in children with ASD have been reported and associations between specific microbial genera and some symptoms of ASD have been described (Tomova et al., 2015). Specifically, a lower ratio of Bacteroidetes-to-Firmicutes and a higher abundance of *Clostridium* and *Desulfovibrio* were positively associated with severity of ASD symptoms (Tomova et al., 2015). In addition, differences in microbial products, such as volatile chain fatty acids (VFA), between children with ASD and unaffected controls have also been observed (Wang et al., 2012).

Achieving adequate dietary intake is challenging in children with ASD and some nutrient deficiencies have been identified (Ledford and Gast, 2006; Liu et al., 2016). Picky eating, food selectivity and food refusal are common behaviors observed in children with ASD and some children might eat as little as 5 foods (Cermak et al., 2010). Picky eating behaviors might be a manifestation of repetitive behavior patterns, ritualistic or externalizing behaviors (Johnson et al., 2014). Others suggest that picky eating might be a reflection of the child's resistance to change, inflexibility, sensory sensitivities, inadvertent reinforcement of negative mealtime behaviors, GI problems and oral motor delay (Cermak et al., 2010; Johnson et al., 2014).

Diet-induced changes in microbiota composition can lead to increased risk of developing certain diseases (e.g., inflammatory bowel diseases), whereas a healthier long-term dietary pattern may be more beneficial in promoting a microbial profile that could potentially protect against diseases (Albenberg and Wu, 2014). Research in animal models has shown that diet-induced changes in the GI microbiota could induce behavioral changes (Li et al., 2009; Pyndt Jørgensen et al., 2014). For example, rodents fed a meat-containing diet had an increased microbial diversity as well as improved working and reference memory compared to animal fed a regular chow diet (Li et al., 2009). Other studies have shown that high calorie diets resulted in poorer cognitive flexibility in mice, which coincided with changes in the GI microbiota composition (Magnusson et al., 2015).

Despite the accumulating evidence linking microbiota composition and metabolism with diet, coupled with the knowledge that dietary diversity is often limited in children with ASD, few studies have concurrently assessed dietary intake and microbiota in this population. Therefore, the goal of this study was to investigate the impact of dietary patterns and nutrient intake on the GI microbiota and VFA in children with ASD and to test whether specific dietary factors could influence the relationship between the GI microbiota and some symptoms of ASD. We hypothesized that children with ASD with a dietary pattern high in fruit, vegetables and grains will harbor a microbiota that is associated with less severe symptoms of ASD. We further hypothesized that healthy food groups will have a more favorable effect on the relationship between specific bacterial taxa and symptoms of ASD compared to unhealthy food options.

## Materials and methods

### Participants and questionnaire

Children diagnosed with ASD between 2 and 7 years-of-age (ASD; *n* = 26) were recruited from sites across Champaign-Urbana and surrounding areas between April 2016 and October 2017. Age- and sex-matched unrelated control subjects (CONT; *n* = 32) were recruited in the Champaign-Urbana area. All subjects were free from functional digestive disorders, had not used antibiotics, probiotics or prebiotic in the 3 months prior to enrollment in the study, did not take any routine medications and did not follow any special diet (e.g., gluten-free/casein-free diet). Parents completed an online questionnaire, including questions regarding their child's age, gender, mode of delivery, early feeding practices, nutritional supplement use, height and weight. Parents also answered questions on their perception of child's feeding problems based on information obtained from previously published results [“Do you consider your child to be a picky eater” (Taylor et al., 2015), “Does your child have a repetitive eating pattern (i.e., likes to eat the same foods)” (Cornish, 1998) and “Does your child currently include more than 20 foods in his or her diet” (Nadon et al., 2011)]. The height, weight and BMI of the participants were converted to percentiles according to the CDC growth charts for both male and female participants. Participants provided oral assent and their legal guardians provided written consent in accordance with the ethical standards of the Institutional Review Board of the University of Illinois at Urbana-Champaign.

### Fecal sample collection

A freshly-voided morning stool sample was collected from each subject for the analysis of the fecal microbiota and VFA concentrations. Stool samples were collected into a plastic commode specimen collection system (Fisher Scientific, Waltham, MA) or directly from the diaper. Parents were provided with gloves and a sterile spoon to transfer ~5–10 g of fecal material into a sterile 50 mL conical tube. All samples were immediately placed in the participant's freezer (−20°C) until transported to the laboratory. All samples were stored in the laboratory at −80°C prior to analysis.

### DNA extraction and 16S rRNA sequencing and analysis

Microbial DNA was extracted from stool using a bead beating method followed by a combination of QIAamp Fast DNA Stool Mini Kit (Qiagen, Valencia, CA) and the FastPrep-24 System (MP Biomedicals, Carlsbad, CA) as previously described (Li et al., 2012). DNA concentration was measured using the Nanodrop 2000 Spectrophotometer (ThermoFisher Scientific, Waltham, MA). Amplification of the V3 to V4 regions (ca. 430 bp) of 16S rRNA gene was performed using dual-index paired-end sequencing approach using primers F357 and R805 (Klindworth et al., 2013). The AccuPrime™ Taq DNA Polymerase System (Life Technologies, Grand Island, NY) was used for PCR amplification in a DNAEngine (Bio-Rad, Hercules, CA). The amplicons were mixed in equimolar concentration and sequenced at the W. M. Keck Center at the University of Illinois, Urbana-Champaign. Paired-end sequencing (2 × 300 bp) was performed with an Illumina MiSeq (Illumina, Inc., San Diego, CA) using version 3 chemistry.

The 16S rRNA sequences were processed and analyzed using the QIIME 1.9.1 bioinformatics package (Caporaso et al., 2010; Bokulich et al., 2013). Sequences were demultiplexed and clustered into operational taxonomic units (OTUs) using closed-reference OTU picking with default parameters against the Greengenes 13_8 reference OTU database at a 97% similarity level. Singletons and OTUs with an abundance lower than 0.005% were removed prior to rarefying to a sampling depth of 49,446 sequences per sample for subsequent analysis. α- and β- diversity were calculated using QIIME. Taxonomy summary was performed using the core diversity script in QIIME.

### Real-time polymerase chain reaction

Bacterial genomic DNA was analyzed for total bacteria, *Lactobacillus* spp., *Bifidobacterium* spp., *Prevotella, Clostridium perfringens*, and *C. difficile*. In addition, bacterial genomic DNA was analyzed for the presence of the propionate-producing gene methylmalonyl-CoA decarboxylase (mmdA) and the butyrate-producing gee butyryl-CoA:acetate CoA acyltransferase (BCoAT). Primer/probe sequences and annealing temperatures are shown in Supplemental Table 1. Real-time qPCR was performed in Quant Studio 6 and 7 Flex Real-Time PCR System (Thermo Fisher Scientific) using SYBR Green assays. Each reaction contained 5 μL of 2x Power SYBR Green PCR Master Mix (Applied Biosystems), 1 μL of bovine serum albumin (New England Biolabs, Ipswich, MA) at 1 mg/mL (final concentration 100 μg/ml), 0.5 μmol/L of each primer and 1 μL of water, 8 μL of PCR mix and 2 μL of sample containing 10 ng of DNA (*Lactobacillus* spp., *Bifidobacterium* spp., *Prevotella, C. perfringens, C. difficile*), 0.5 ng of DNA (total bacteria) or 20 ng (mmDA, BCoAT) were plated on a MicroAmp Optical 384–well reaction plate (Applied Biosystems). The cycling conditions were 50°C for 2 min, 95°C for 10 min, 40 cycles of 95°C for 15 s, primer-specific annealing temperature for 20 s and 72°C for 45 s. Standard curves (5 x 10^9^–5 x 10^7^ gene copies per reaction) were prepared using purified PCR 4 TOPO-TA plasmids (Life Technologies) containing 16S rRNA genes of *Eubacterium hallii* 27751 (total bacteria), *Lactobacillus rhamnosus* 53103 (Lactobacillus spp.), *Bifidobacterium longum* 15007 (Bifidobacterium spp.), *Prevotella ruminicola* 19189 (Prevotella), *C. perfringens* 13124 (C. perfringens), and *C. difficile* 9689 (C. difficile). mmdA and BCoAT genes were quantified in parallel with a universal 16S rRNA gene. Data was analyzed using QuantStudio Design Analysis Software 1.3 (Thermo Fisher Scientific). Results of bacteria are presented as the log_10_ of the number of copies per gram of wet sample and mmdA or BCoAT over total 16S rRNA.

### VFA analysis

Sample preparation for VFA analyses was performed as follows as previously described (Li et al., 2012). The VFA concentrations were analyzed by a Hewlett-Packard 5890A Series gas chromatograph and a glass column (180 cm 3 4 mm i.d.), packed with 10% SP-1200/1% H3PO4 on 80/100 mesh Chromosorb WAW (Supelco, SigmaAlderich, St. Louis, MO). Oven temperature, detector temperature, and injector temperature were set at 1,258, 1,758, and 1,808°C, respectively. VFA production was calculated as VFA concentrations of substrate-containing tubes minus the VFA content of blank tubes divided by substrate weight expressed on a dry matter basis.

### ASD symptoms severity assessment

Parents were asked to complete the Pervasive Development Disorder Behavior Inventory Screening Version (PDDBI-SV) (PAR, Inc., Lutz, FL) in order to assess the social deficit symptom severity (Cohen, 2011). The PDDBI-SV is an 18-item parent questionnaire developed for children ages 18 months−12.5 years. Nine questions from the Social Pragmatic Problems domain and 9 questions from the Social Approach Behaviors from the original PDDBI were used to develop the screening version. Each question has 5 response options that are rated on a Likert scale. In the Social Pragmatic Problem domain, the answers are scored according to 0 “Does not show behavior,” 1 “Rarely shows behavior,” 2 “Sometimes/Partially shows behavior,” 3 “Usually/typically shows behavior,” and “Don't understand.” In the Social Approach Behaviors Domain, the answers are reverse-scored. The answers are scored by summing the ratings to yield 1 composite score, the Social Deficit (SOCDEF) score. As the SOCDEF score increases, social communication skills worsen and challenging behaviors increase.

### Assessment of gastrointestinal symptoms and stool consistency

The severity of GI symptoms (constipation, diarrhea, stool smell, flatulence and abdominal pain) was assessed was on a scale from 0 to 2 using an adapted version of the GI Severity Index (Adams et al., 2011). Scores for each item were summed to determine an overall severity score.

Additionally, average stool consistency was assessed using the Bristol Stool Chart (Lewis and Heaton, 1997).

### Dietary intake quantification

Dietary intake was measured using a 3–day food diary and a revised version of the semi-quantitative Youth and Adolescent Food Frequency questionnaire (YAQ) as previously described (Berding et al., 2018). To monitor short-term nutrient intake, parents completed a dietary food record for their child on the 3 days prior to stool sample collection. The food diary data were analyzed using the Nutrition Data System for Research (NDSR, Minneapolis, MN, 2014) software to assess nutrient intake and for comparison to recommended intakes (i.e., Recommended Daily Allowance, Adequate Intake).

To estimate the number of servings of any food group, each response in the YAQ was converted to the corresponding frequency factor and summed over all the food items to get the average servings of a specific food group per day.

### Statistics

All data were analyzed using SAS 9.4 (SAS Institute, Cary, NC). Descriptive statistics (means and frequencies) were generated for all demographic and epidemiologic variables of study participants. Data are expressed as mean ± SD. Level of significance was set at *P* ≤ 0.05 and *P* ≤ 0.10 was considered a trend.

Differences in microbial community structure were evaluated with principal coordinate analysis (PCoA) and permutational multivariate analysis (PERMANOVA) of variance using weighted and unweighted UniFrac distance in the QIIME software. Differences between the groups in α-diversity (Chao1, Shannon Index, Simpson Index and observed OTUs), relative abundance of individual phyla, families, orders and genera, VFA and nutrient intake were analyzed using generalized linear mixed models. Model fit was assessed using the Chi-sqaure-to-df ratio. Values < 2 indicated an appropriate model fit. Factors known to influence the microbiota composition including age, gender, BMI, height, weight, season of sample collection and dietary fiber intake were included as co-variates in each model. Categorical variables were analyzed for significant differences between the groups using the Fischer's Exact test. For the ASD group, dietary patterns data derived from the YAQ were analyzed using Principal Component Analysis and Factor Analysis with Varimax rotation as previously described (Berding et al., 2018). Differences in microbiota composition, VFA concentrations and nutrient intake between participants in the ASD group falling above or below the median for each dietary pattern were analyzed using generalized linear mixed models.

## Results

### Participant characteristics

The study population demographics and characteristics for each group are shown in Table 1. Parents of children in CONT were more likely to have higher education (*p* = 0.001) and participants in the CONT group were more likely to be exclusively breast-fed (*p* = 0.03) compared to the ASD group. Additionally, participants in the CONT tended (*p* = 0.08) to be slightly taller. There was no difference in age, gender, race/ethnicity, nutritional supplement use, route of birth, gestational age, or antibiotic use in early life between the groups. Likewise, parental marital status, annual household income as well as health care coverage did not differ between the groups. Regarding their eating behavior, children with ASD were less likely (*p* = 0.0007) to consume more than 20 foods in their diet compared to the CONT group. Presence of GI symptoms was reported in 69% of children with ASD and 46% of unrelated control children. GI symptoms severity was about twice as high (*p* = 0.002) in the ASD group compared to CONT. Specifically, children with ASD had higher scores for stool smell (*p* = 0.006); flatulence (*p* = 0.04) and abdominal pain (*p* = 0.07). Likewise, stool consistency measured by the Bristol stool chart differed significantly (*p* = 0.05) between the groups.

**Table 1:** Demographic and health characteristics of study participants.

**Characteristic**	**ASD (*n* = 26)**	**CONT (*n* = 32)**
Age (years)	4.1 ± 1.6	4.8 ± 1.8
Gender (*n*)		
Male	19	19
Female	7	13
Race/ethnicity [*n*(%)]		
Caucasian	15 (68%)	19 (63%)
Asian	1 (5%)	5 (17%)
Black of african american	3 (14%)	3 (10%)
Hispanic or latino	1 (5%)	1 (3%)
Other	2 (9%)	2 (7%)
Parent level of education		
High school	0	0^*^
Some college or technical school	6 (30%)	1 (3%)^*^
College graduate	11 (55%)	10 (33%)^*^
Post-graduate work	4 (20%)	19 (63%)^*^
Weight (kg)	20.5 ± 7.8	21 ± 0.7
Height (meters)	1.07 ± 0.1	1.10 ± 0.15^†^
Mean BMI and percentile (BMI-for-age)		
Male	16.8 (70th)	16.3 (75th)
Female	15.1 (50th)	16.8 (82nd)
Nutritional supplement use [*n* (%)]		
Yes	12 (46%)	14 (44%)
No	14 (54%)	18 (56%)
Route of birth [*n* (%)]		
Vaginal	11 (42%)	18 (56%)
Planned C-section	6 (23%)	4 (13%)
Emergency C-section	9 (35%)	10 (31%)
Gestational age [*n* (%)]		
<37 weeks	3 (12%)	2 (6%)
37–42 weeks	19 (73%)	27 (84%)
>42 weeks	4 (15%)	3 (9%)
Early feeding mode [*n* (%)]		
Breast-fed only	5 (20%)	17 (53%)^*^
Breast-fed in combination with formula	17 (65%)	13 (41%)^*^
Formula only	4 (15%)	2 (6%)^*^
Antibiotics use in early life [*n* (%)]		
Yes	4 (15%)	2 (6%)
No	22 (85%)	30 (94%)
Picky eater		
Yes	13 (50%)	10 (31 %)^†^
No	13 (50%)	22 (69%)^†^
More than 20 foods in diet [*n* (%)]		
Yes	12 (46%)	29 (90%)^*^
No	14 (54%)	3 (10%)^*^
Repetitive eating pattern		
Yes	15 (54%)	13 (36%)
No	11 (46%)	19 (64%)
GI severity score^a^	2 (0–3)	0 (0–1)^*^
Constipation	1 (0–2)	0 (0–1)
Diarrhea	0	0
Stool smell	0 (0–1)	0^†^
Flatulence	0 (0–1)	0^†^
Abdominal pain	0 (0–1)	0^†^
Stool consistency (*n*)^b^		
Type 1 (separate hard lumps)	0	1^*^
Type 2 (sausage shaped but lumpy)	5	3^*^
Type 3 (sausage-shaped with cracks on surface)	5	13^*^
Type 4 (smooth and soft)	12	15^*^
Type 5 (soft blobs)	2	0^*^
Type 6 (mushy)	2	0^*^
Type 7 (watery)	2	0^*^

a *GI severity scores were derived from the GI severity index (possible range 0–6)*.

b *Stool consistency was measured using the Bristol Stool chart*.

Data expressed as mean ± SD or median (IQR); within same segment and row, different from CONT at

* p ≤ 0.05 and

† *≤ 0.1*;

*ASD, Autism Spectrum Disorder group; CONT, unaffected control group*.

### Microbiota composition and VFA concentrations

**Figure 1:**
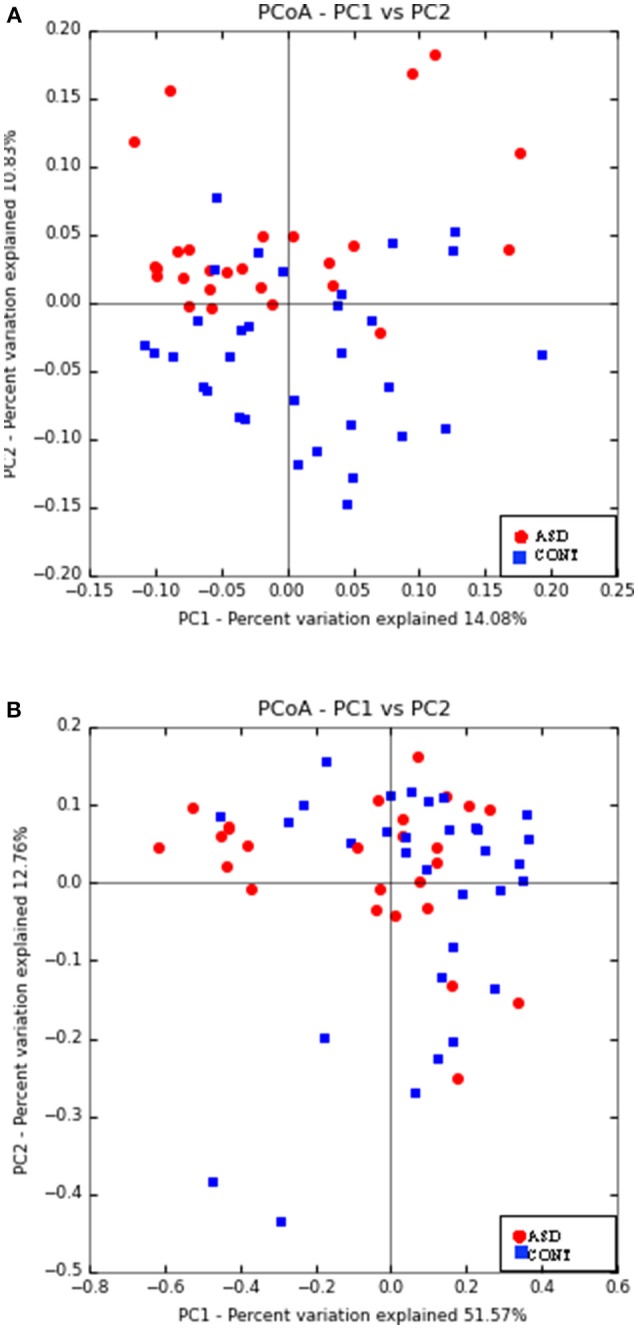
Principal co-ordinate analysis based on unweighted UniFrac **(A)** and weighted UniFrac Distance **(B)** generated from fecal samples of children with ASD (ASD) and unaffected controls (CONT). PERMANOVA analysis indicated that overall bacterial communities differed between ASD and CONT group (*p* = 0.02) based on unweighted UniFrac, but not on weighted UniFrac distance.

Principal Coordinate Analyses (PCoA) of weighted and unweighted UniFrac are shown in Figure 1. PERMANOVA analysis indicated that overall bacterial communities differed between ASD and CONT group (*p* = 0.02) based on unweighted UniFrac, but not on weighted UniFrac distance. Fecal α-diversity tended to differ between the groups when measured as observed OTUs (*p* = 0.08). There were no differences on other measures of α-diversity (Chao 1 Index, Shannon Index, Simpson Index, Phylogenetic Diversity Whole Tree) between the groups.

To identify which specific bacteria differed between the groups, the sequences were classified using the Greengenes Database (Supplemental Table 2). At the phyla level, children with ASD had a lower abundance of Bacteroidetes (*p* = 0.07), but higher abundance of Firmicutes (*p* = 0.03) compared to CONT. Additionally, the abundance of Clostridiales (*p* = 0.07) was higher, whereas the abundance of Streptophyta (*p* = 0.08) was lower in children with ASD compared to CONT. On the family level, children with ASD had significantly higher abundance of Coriobacteriaceae (*p* = 0.04), Clostridiaceae (*p* = 0.07) and Peptostreptococcaceae (*p* = 0.05), but a lower abundance of Rikenellaceae (*p* = 0.005) compared to CONT children. On the genera level, increased abundances of Clostridiaceae *Clostridium, SMB53, Blautia*, and *Roseburia*, but decreased abundances of *Butyricimonas, Butyrivibrio, Faecalibacterium, Dialister*, and *Bilophila* were observed in children with ASD. The abundances of *Bifidobacterium* spp. (*p* = 0.04) and *C. perfringens* determined by qPCR were higher (*p* = 0.009) in the CONT group compared to the ASD group.

**Figure 2:**
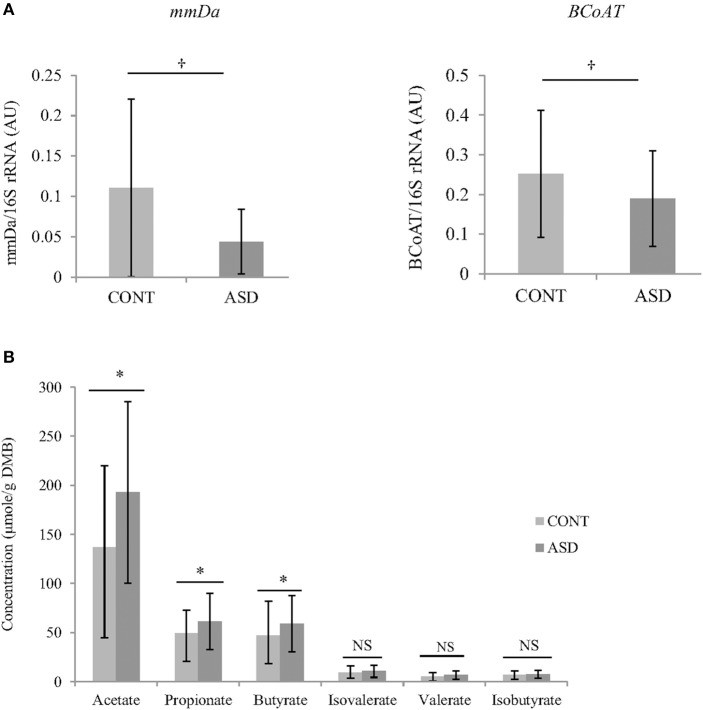
**(A)** Relative mRNA abundance of butyrate-producing gene BCoAT and propionate-producing gene mmDA in feces of ASD and CONT children measured by qPCR. **(B)** Differences in VFA concentrations between CONT and ASD. ^*^p ≤ 0.05; ^†^*p* ≤ 0.1; Data is expressed as mean ± SD; mmDA, methylmalonyl-CoA decarboxylase; BCoAT, butyryl-CoA:acetate CoA acyltransferase; ASD, Autism Spectrum Disorder group; CONT, unaffected control group; VFA, volatile fatty acids; DMB, dry matter basis.

In CONT children, a trend for greater relative BCoAT (*p* = 0.09) and mmDA (*p* = 0.07) gene was observed compared to ASD children. Higher concentration of acetate (*p* = 0.02), propionate (*p* = 0.04) and butyrate (*p* = 0.03) were observed in the ASD group compared CONT. There was no statistically significant differences in the concentrations of valerate, isovalerate and isobutyrate (Figure 2).

### Nutrient intake and food groups

Nutrient intakes derived from the 3-day food diary and food group intakes derived from YAQ are summarized in Supplemental Table 3. Overall, there were no differences in energy or macronutrient intake between the ASD and CONT group. Total dietary fiber intake did not differ between the groups; however, children with ASD tended (*p* = 0.09) to have a lower intake of insoluble dietary fiber and pectin intake, than CONT. In regards to vitamins and minerals, the only difference was observed in intake of vitamin C, with the ASD group having lower (*p* = 0.01) intakes compared to CONT children. Regarding food group intake, children with ASD ate fewer servings per day of dairy (*p* = 0.05), and tended to consume more snacks (*p* = 0.09) and sweets (*p* = 0.1) per day compared to CONT children.

### Variables predicting ASD score

In order to explore the relationship between the microbiota and metabolic products, stepwise regression was used to analyze whether individual microbiota or microbial products could predict the SOCDEF scores. Due to the high number of variables, stepwise regression was used to assist in identifying potential independent variables for predicting SOCDEF scores. α-diversity, relative concentration of BCoAT and mmDA as well as VFA concentrations and GI symptom severity did not reach the significance level. In regards to bacterial family, order, phyla and genera Peptostreptococcaceae, *Lactobacillus, Dialister*, and *Faecalibacterium* were identified as significant predictors for SOCDEF score.

Model adequacy was tested for each model including the independent variables identified through stepwise regression.

After quality control, the remaining microbiota in the model, namely Peptostreptococcaceae and *Faecalibacterium* produced an adjusted *R*^2^ of 0.36 [*F*_(5, 18)_ = 5.96; *p* = 0.009] for the prediction of SOCDEF score. Thereby, Peptostreptococcaceae (β = 0.33) and *Faecalibacterium* (β = 0.63) positively predicted SOCDEF scores. A summary of the fitted models can be found in Table 2. Age, gender, season, height, weight and BMI were included as co-variates in the model.

**Table 2:** Regression analysis model showing fecal bacterial taxa predicting social deficit scores.

**Variable**	**Parameter estimate B**	**Standardized estimate β**	**Standard error**	***t*-value**	***P*-value**	**Squared semi-partial corr type II**
Intercept	43.5		3.02	14.41	< 0.0001	.
*Faecalibacterium*	0.9	0.63	0.27	3.39	0.003	0.35
Peptostreptococcaceae	14.1	0.33	7.96	1.77	0.09	0.09

*Bacteria expressed as relative abundance derived from 16S rRNA sequencing. SOCDEF scores, social deficit scores derived from PDDBI-SV; adjusted R^2^ 0.36 [F_(5, 18)_ = 5.96; p = 0.009]*.

### Picky eating behavior and repetitive eating pattern are associated with microbiota composition in children with ASD

In order to analyze potential differences in the gut microbiota composition based in eating behavior of children with ASD, microbiota composition and VFA concentration between children with ASD who were dichotomized based on parent report of picky eating and repetitive eating patterns was investigated. No differences in SOCDEF scores based on reported picky eating behavior or repetitive eating pattern were found. However, differences in the nutrient and food group intake as well as microbiota composition based on picky eating behavior and repetitive eating patterns was observed (Table 3).

**Table 3:** Differences in nutrient intake and microbiota composition between children with ASD characterized by picky eating behavior, including 20 foods in diet and repetitive eating pattern.

**Variable**	**Yes (*n* = 11)**	**No (*n* = 15)**
**(A) PICKY EATING BEHAVIOR**		
Age	4.6 ± 1.5	3.6 ± 1.3
Gender		
Female	2	5
Male	9	10
Weight	47.4 ± 22.4	41.7 ± 7.3
Height	3.5 ± 0.5	3.4 ± 0.4
BMI	17.1 ± 3.1	17.4 ± 1.9
SOCDEF T-Score	54 ± 11	51 ± 8
GI severity score	3 (1.5–3)	1.5 (0–3)
Constipation	1 (0–2)	0.5 (0–2)
Diarrhea	0 (0–0)	0 (0–0)
Stool smell	0 (0–1)	0 (0–1)
Flatulence	0 (0–0.5)	0.5 (0–1.5)
Abdominal pain	0.5 (0–1)	0 (0–0)^*^
**NUTRITION**		
Nutrient intake		
Total fat	45 (35–52)	55 (45–64)^†^
Monounsaturated fatty acids	13.5 (11.5–17.6)	18.2 (16.9–24.4)^†^
Food groups		
Juice	0.9 (0.8–1.0)	0.08 (0–0.8)^*^
Protein foods	0.8 (0.3–1.7)	1.6 (0.9–2.2)^†^
**BACTERIAL FAMILY**		
Coriobacteriaceae	0.31 (0.02–1.12)	0.02 (0.002–0.05)^*^
EtOH8	0 (0–0.06)	0 (0–0)^†^
**BACTERIAL GENUS**		
*Bacteroides*	15.5 (8.6–29.22)	37.3 (29.6–47.0)^†^
*Ruminococcus*	3.05 (1.9–6.9)	0.9 (0.17–2.1)^†^
*Holdemania*	0.01 (0.002–0.02)	0.001 (0–0.007)^*^
*Phascolarctobacterium*	0.03 (0.0008–0.09)	0.002 (0.0008–0.36)^†^
**VFA**		
Isobutyrate	7.82 (5.51–13.54)	5.78 (3.1–7.6)^†^
Isovalerate	11.4 (6.3–20.4)	7.4 (4.5–11)^†^
**Variable**	**Yes (*****n*** = **12)**	**No (*****n*** = **14)**
**(B) 20 FOODS IN DIET**		
Age	4.1 ± 1.5	4.3 ± 1.5
Gender		
Female	3	4
Male	9	9
Weight	38.9 ± 7.9	50.5 ± 21.8
Height	3.4 ± 0.5	3.6 ± 0.4
BMI	15.9 ± 1.5	18.3 ± 2.9^*^
SOCDEF T-Score	53 ± 8	52 ± 11
GI severity score	1 (0–2)	3 (2–3)^*^
Constipation	0 (0–2)	1 (0–2)
Diarrhea	0 (0–0)	0 (0–0)
Stool smell	0 (0–0)	0 (0–1)
Flatulence	0 (0–0)	1 (0–2)^*^
Abdominal pain	0 (0–0)	1 (0–1)^†^
**NUTRITION**		
Nutrient intake		
Pectins	1.4 (1.1–2.8)	1.2 (1.06–1.6)^†^
Vitamin C	54 (33–98)	31 (24–41)^†^
Niacin	16 (13–21)	12 (10–14)^*^
Vitamin B6	1.2 (1.04–2.0)	1.14 (0.88–1.29)^†^
Folate	243 (189–401)	226 (201–265)^†^
Selenium	67 (60–80)	58 (45–84)^†^
Added sugars	29 (24–39)	36 (22–47)^*^
**BACTERIAL PHYLA**		
Actinobacteria	1.7 (0.7–2.9)	6.3 (3.1–13.6)^*^
Bacteroidetes	55.2 (45.6–60.5)	39.7 (13.1–48.4)^*^
Cyanobacteria	0.0007 (0–0.007)	0 (0–0)^†^
**BACTERIAL ORDER**		
Streptophyta	0.0007 (0–0.007)	0 (0–0)^*^
**BACTERIAL FAMILY**		
Coriobacteriaceae	0.03 (0.005–0.07)	0.11 (0.007–0.9)^†^
Clostridiales	2.66 (1.55–3.37)	3.61 (2.66–7.17)^*^
**BACTERIAL GENUS**		
*Bifidobacterium*	0.96 (0.37–2.52)	2.58 (1.06–6.00)^†^
*Collinsella*	0.02 (0.006–0.21)	1.21 (0.31–4.2)^†^
*Eggerthella*	0.1 (0.05–0.37)	0.02 (0.01–0.05)^*^
*Bacteroides*	40.2 (25.7–55.9)	25.8 (8.6–36.0)^†^
*Lactobacillus*	0 (0–0)	0.001 (0–0.01)^†^
*Dialister*	0.62 (0.12–1.29)	0.008 (0.003–0.01)^*^
**VFA**		
Valerate	4.16 (1.08–8.6)	6.8 (4.7–8.1)^†^
**Variable**	**Yes (*****n*** = **17)**	**No (*****n*** = **9)**
**(C) REPETITIVE EATING BEHAVIOR**		
Age	4.4 ± 1.6	3.7 ± 1.1
Gender		
Female	3	4
Male	13	6
Weight	49 ± 20.8	38.7 ± 7.5
Height	3.6 ± 0.5	3.9 ± 0.4
BMI	18.1 ± 2.8^*^	15.8 ± 1.4
SOCDEF T-Score	54 ± 8	51 ± 10
GI severity score	3 (2–3)	0.5 (0–2)^*^
Constipation	1 (0–2)	0 (0–2)
Diarrhea	0 (0–0)	0 (0–0)
Stool smell	0 (0–1)	0 (0–1)
Flatulence	1 (0–2)	0 (0–0)^*^
Abdominal pain	0.5 (0–1)	0 (0–0)^*^
**NUTRITION**		
Nutrient intake		
Pectins	1.2 (1.0–1.6)	1.6 (1.2–2.9)^†^
Vitamin C	33 (25–43)	84 (33–99)^*^
Potassium	1340 (1034–1982)	1844 (1475–2394)^†^
Copper	0.64 (0.49–0.75)	0.94 (0.75–1.24)^*^
**BACTERIAL PHYLUM**		
Actinobacteria	5.8 (1.5–14.5)	2.7 (1.0–3.04)^*^
Verrucomicrobia	0.09 (0.02–0.72)	3.99 (0.02–6.7)^†^
Cyanobacteria	0 (0–0)	0.001 (0–0.009)^†^
**BACTERIAL ORDER**		
Clostridiales	3.6 (2.7–7.4)	1.9 (0.9–3.2)^†^
Streptophyta	0 (0–0)	0 (0–0.003)^*^
**BACTERIAL FAMILY**		
Coriobacteriaceae	0.08 (0.02–0.76)	0.01 (0.002–0.39)^*^
**BACTERIAL GENUS**		
*Adlercreutzia*	0.06 (0.04–0.19)	0.006 (0.001–0.39)^†^
*Collinsella*	0.96 (0.14–4.26)	0.08 (0.005–0.25) ^†^
*Eggerthella*	0.03 (0.01–0.07)	0.14 (0.06–0.56)^†^
*Butyrivibrio*	0.01 (0.005–0.03)	0.14 (0.003 (0–0.01)^*^
*Dialister*	0.009 (0.003–0.44)	0.56 (0.01–0.9)^†^
*Coprobacillus*	0 (0–0)	0.0004 (0–0.01)^†^
*Akkermansia*	0.09 (0.02–0.72)	3.98 (0.02–6.71)^†^

Data expressed as mean ± SD or median (IQR); all outcome parameters were analyzed for each feeding behavior; only outcomes with significant differences are shown; within same segment and row, different at

* p ≤ 0.05 and

† *≤ 0.1*.

#### Picky eating behavior

Children with ASD who were described as picky eaters by their parent had higher abdominal pain scores compared to non-picky eaters. In regard to nutrition, children described as picky eaters had lower intakes of total fat, monounsaturated fatty acids, and protein foods, but higher intakes of juice. Regarding the microbiota composition, children with picky eating behavior had higher abundance of Coriobacteriaceae and EtOH8. On the genera level, children described as picky eaters had higher abundance of *Ruminococcus* and *Holdemania*, but lower relative abundance of Bacteroides and *Phascolarctobacterium*. Lastly, higher concentrations of isobutyrate and isovalerate were observed in children with ASD described as picky eaters.

#### Including 20 or fewer foods in the diet

Children with ASD who regularly consumed fewer than 20 foods in their diet had a higher BMI as well as higher scores for total GI severity, flatulence and abdominal pain compared to children with ASD who consumed more than 20 foods in their diet. In regard to their nutritional intake, children eating 20 foods or less had lower intakes of pectin, vitamin C, niacin, vitamin B6, folate, and selenium, but higher intakes of added sugars. Regarding the microbiota composition, children with 20 foods or less in the diet had higher levels of Actinobacteria, Coriobacteriaceae, Clostridiales, *Bifidobacterium, Collinsella, Lactobacillus*, and *Acidaminococcus*, but lower abundances of Bacteroidetes, Cyanobacteria, *Eggerthella*, Bacteroides, *Dialister* and *Anaerotuncs*. Concentrations of valerate tended to be higher in children with ASD who included less than 20 foods in their diet.

#### Repetitive eating pattern

Children with ASD who displayed repetitive eating patterns also had a higher BMI and higher scores in total GI severity scores, flatulence and abdominal pain. Nutritionally, children with repetitive eating patterns had lower intakes of pectin, vitamin C, potassium, and copper. At the bacterial order level, lower abundance of Streptophyta, but higher abundances of Clostridiales were observed in children with repetitive eating patterns. Additionally, children with ASD and repetitive eating patterns had higher abundance of Coriobacteriacea and Actinobacteria, but lower abundance of Verrucomicrobia and Cyanobacteria. Lastly, at the genus level children with repetitive eating behaviors had higher levels of *Collinsella* and *Butyrivibrio*, but lower abundance of *Adlercreutzia, Eggerthella, Dialister, Coprobacillus*, and *Akkermansia*.

### Correlation between food groups and microbiota abundance

**Figure 3:**
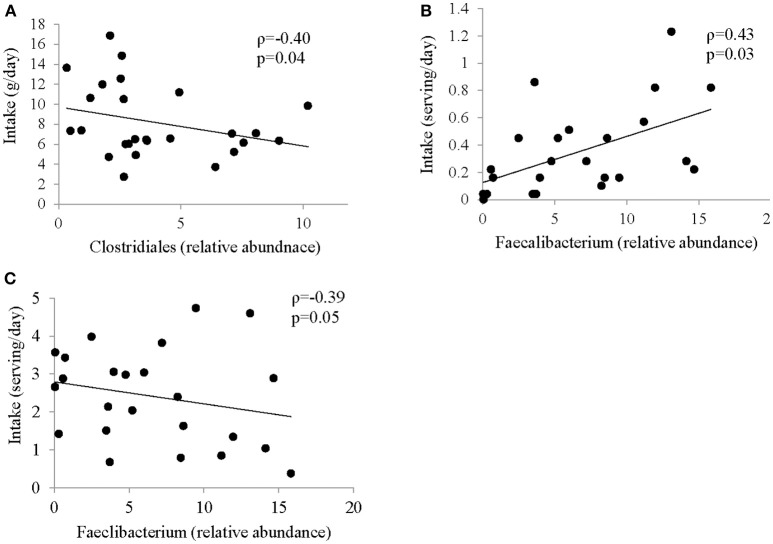
Correlation between intake of specific food group and abundance of bacteria in ASD. Spearman Correlation coefficient for selected food groups and nutrients and abundance of bacteria showing strongest correlation; **(A)** insoluble dietary fiber and Clostridiales; **(B)** Fried Food and *Faecalibacterium*; **(C)** Fruit and *Faecalibacterium*; all correlations are shown in Supplemental Table 4.

Several associations between dietary factors and microbiota composition in both groups were identified. The strongest correlations for the ASD group are shown in Figure 3. All other correlations can be found in Supplemental Table 4. Intake of insoluble dietary fiber was negatively correlated (ρ = −0.4; *p* = 0.04) with abundance of Clostridiales. *Faecalibacterium* abundance was positively correlated with servings per day of fried food (ρ = 0.43; *p* = 0.0), but negatively correlated with servings per day of fruit (ρ = −0.39; *p* = 0.05).

### Dietary patterns, participant characteristics, microbiota composition, and VFA concentration children with ASD

Using factor analysis, two distinct dietary patterns for the ASD group were identified. Dietary Pattern 1 (DP1) was characterized by an intake of vegetables, legumes, nuts and seeds, fruit, starchy vegetables, grains, juice and dairy. Dietary Pattern 2 (DP2) was characterized by an intake of fried foods, Kid's meals, condiments, snacks, starchy foods and protein foods. Refined carbohydrates was present in both dietary patterns, but was more associated with DP 1. Fish, sweets and sweetened beverages were not significantly associated with either dietary pattern. The factor loading matrix for children with ASD can be found in Table 4.

**Table 4:** Factor loading matrix for dietary patterns in children with ASD.

**Food group**	**Dietary pattern 1**	**Dietary pattern 2**
Vegetables	0.88^*^	0.02
Starchy vegetables	0.79^*^	0.16
Legumes, nuts and seeds	0.78^*^	0.08
Fruit	0.73^*^	0.13
Grains	0.56^*^	−0.002
Juice	0.45^*^	0.13
Dairy	0.36^*^	0.13
Refined carbohydrates	0.72^*^	0.50^*^
Fried foods	0.13	0.67^*^
Kids Meals	−0.07	0.67^*^
Condiments	0.14	0.64^*^
Protein foods	0.23	0.62^*^
Snacks	−0.33	0.59^*^
Starchy foods	0.32	0.42^*^
Fish	−0.03	0.21
Sweets	0.11	0.05
Sweetened beverages	−0.29	0.26

* *Factor loading >0.35 is considered to be a major contributor to the overall dietary pattern; Food groups in which factor loadings are >0.35 for both dietary pattern are assigned to dietary pattern for which food group has highest factor loading contribution*.

Participant characteristics of the ASD group were dichotomized by category of factor score (above or below the median) in order to analyze differences in participant characteristics, nutrient intakes, VFA concentration and bacterial abundance based on long-term dietary pattern (Tables 5–7). No differences in participant characteristics (e.g., age, gender etc.) and SOCDEF scores based on dietary patterns were observed.

**Table 5:** Participant characteristics by scores above or below the median in children with ASD.

	**Dietary pattern 1**		**Dietary pattern 2**	
Characteristic	Above median	Below median	Above median	Below median
	(*n* = 13)	(*n* = 13)	(*n* = 13)	(*n* = 13)
Age	3.8 ± 1.4	4.4 ± 1.6	4.4 ± 1.5	4 ± 1.4
Gender (*n*)				
Male	11	9	10	9
Female	2	4	3	4
BMI (kg/m^2^)	16.6 ± 2.2	17.7 ± 2.9	17.5 ± 3	16.8 ± 2.1
Race (*n*)				
Caucasian	8	7	8	7
Asian	0	1	1	0
Black of african american	2	1	1	2
Hispanic or latino	0	1	1	0
Other	2	0	0	2
Current nutritional supplement use (*n*)	6	5	6	5
Picky eater (*n*)	5	6	6	5
>20 foods in diet (*n*)	8	4	4	8
Repetitive eating pattern (*n*)	7	9	9	7
GI severity index	1 (0–3)	3 (2–3)^*^	2 (1.5–3)	2 (0–4)
Constipation	0 (0–1)	2 (0–2)^*^	1.5 (0–2)	0.5 (0–1.5)
Diarrhea	0 (0–0)	0 (0–0)	0 (0–0)	0 (0–0)
Stool smell	0 (0–1)	0 (0–1)	0 (0–0.5)	0 (0–1)
Flatulence	0 (0–0.5)	0.5 (0–2)	0 (0–1)	0 (0–2)
Abdominal pain	0 (0–1)	0 (0–0.5)	0 (0–0.5)	0 (0–1)
Bristol stool chart (*n*)
Type 2	2	4	3	3
Type 3	3	2	3	2
Type 4	5	6	5	6
Type 5	2	0	1	1
Type 6	1	1	1	1
SOCDEF T-Score	52 ± 10	52 ± 9	50 ± 6	54 ± 12

Data are expressed as mean ± SD, median (IQR) or n; values within same dietary pattern and row are significant at

* *p < 0.05 and p < 0.1*.

*GI, gastrointestinal; BMI, body mass index; SOCDEF, social deficit score*.

**Table 6:** Food and nutrient intakes of children with ASD characterized as consuming above or below the median in dietary pattern 1 and dietary pattern 2.

	**Dietary pattern 1**		**Dietary pattern 2**	
	**Above median (*n* = 13)**	**Below median (*n* = 13)**	**Above median (*n* = 13)**	**Below median (*n* = 13)**
**FOOD GROUPS INTAKE (SERVINGS PER DAY)**				
Vegetables	1.6 (0.6–2.3)	0.3 (0.1–0.7)^*^	0.7 (0.2–1.2)	0.6 (0.4–1.6)^†^
Legumes, nuts, seeds	0.4 (0–0.8)	0.1 (0.1–0.2)^*^	0 (0–0.2)	0.1 (0–0.4)^*^
Fruit	2.9 (1.9–3.6)	1.0 (0.5–2.3)^*^	2.3 (0.6–2.9)	1.9 (1.2–3.4)
Refined carbohydrates	1.1 (0.9–1.7)	0.8 (0.4–0.9)^*^	0.9 (0.8–1.4)	0.9 (0.5–1.1)
Sweets	1.2 (0.9–1.8)	2.5 (1.6–3.1)^†^	2.0 (1.4–3.1)	1.7 (0.9–2.3)
Starchy vegetables	0.9 (0.6–1.5)	0.2 (0.1–0.4)^*^	0.3 (0.1–0.5)	0.7 (0.4–1.2)^*^
**NUTRIENT INTAKE**				
Folate (μg)	235 (185–338)	242 (201–294)^†^	218 (193–265)	255 (203–338)
Vitamin E (mg)	6.5 (4.5–9.7)	5.8 (3.4–7.9)^*^	5.8 (4.2–7.8)	6.5 (4.5–9.5)
Vitamin B12 (mg)	2.9 (2.2–3.8)	3.0 (2.1–4.0)^*^	3.0 (2.8–3.4)	2.5 (3.1–4.0)^†^
Vitamin A	534 (442–712)	326 (278–428)^*^	413 (282–565)	442 (278–651)
Vitamin E	14.7 (13.5–16.7)	12.4 (20.6–18.9)	12.4 (12.0–14.7)	16.7 (13.8–20.3)^†^
Niacin	1.2 (0.9–1.3)	1.1 (0.9–1.8)	1.1 (0.9–1.3)	1.3 (0.9–1.9)^†^
Vitamin B6	5.3 (4.3–6.7)	5.0 (4.3–7.4)	4.7 (3.8–5.5)	5.9 (4.8–7.3)^*^
Total Grains	3.9 (2.6–4.8)	4.6 (3.7–5.1)	4.3 (2.8–5.0)	4.6 (3.7–5.4)^†^
**REFINED GRAINS**				
Total dietary fiber (g)	11 (9.8–16.9)	9.6 (7.3–14.8)	9.8 (9.3–12.1)	10.9 (9.2–16.9)
Soluble dietary fiber (g)	4.9 (3.1–5.4)	3.6 (2.4–4.3)	3.3 (2.4–5.2)	3.5 (3.1–5.4)
Insoluble dietary fiber (g)	7.3 (6.4–10.6)	6.3 (4.9–10.5)^†^	6.5 (6.1–7.3)	7.4 (6.0–10.6)
Pectin (g)	1.2 (1.1–1.8)	1.5 (1.1–1.7)	1.3 (1.1–1.7)	1.3 (0.9–1.8)

Data are expressed as median (IQR); values within same pattern and row are significant at

* p < 0.05 and

† *p < 0.1*;

*Only nutrient intake that showed significant difference between the groups are shown*.

**Table 7:** Bacterial abundance and VFA concentrations of participants in ASD group characterized as consuming above or below the median in dietary pattern 1 and dietary pattern 2.

	**Dietary pattern 1**		**Dietary pattern 2**	
**Characteristic**	**Above median (*n* = 13)**	**Below median (*n* = 13)**	**Above median (*n* = 13)**	**Below median (*n* = 13)**
**BACTERIAL ABUNDANCE (% OF SEQUENCES)**				
**Family**
Enterobacteriaceae	0.006 (0.004–0.07)	0.02 (0.008–0.12)^†^	0.17 (0.008–0.12)	0.06 (0.005–0.1)
Barnesiellaceae	0.02 (0.006–1.45)	0.08 (0.006–2.98)	3.0 (0.008–2.85)	0.73 (0.006–0.97)^*^
Streptophyta	0.005 (0–0.02)	0.001 (0–0.004)	0.003 (0–0.004)	0.003 (0–0.005)^†^
**Genera**
*Lactococcus*	0.0007 (0–0.005)	0.008 (0.002–0.02)^*^	0.002 (0–0.008)	0.005 (0.006–0.01)
*Roseburia*	0.28 (0.12–0.41)	0.34 (0.18–0.57)^†^	0.31 (0.14–0.36)	0.38 (0.22–0.51)
*Leuconostoc*	0 (0–0)	0.002 (0–0.002)^†^	0 (0–0)	0 (0–0)
*Ruminococcus*	1.25 (0.89–1.78)	1.60 (0.9–3.83)^†^	1.24 (0.43–1.78)	1.72 (1.21–3.83)
*Alistipes*	0.001 (0–0.02)	0.002 (0–0.01)	0.001 (0–0.04)	0.001 (0–0.004)^†^
**VFA CONCENTRATION (**μ**MOL/G)**				
Acetate	155 (110–255)	173 (131–243)	200 (131–255)	143 (111–246)
Propionate	51 (39–76)	55 (51–90)	68 (51–90)	50 (39–61)^†^
Butyrate	49 (37–74)	66 (42–76)	67 (42–76)	48 (37–74)
Isobutyrate	6.2 (4.25–7.7)	7.8 (4.5–10)	9.4 (6.2–11.5)	5.4 (3.6–7.2)^*^
Valerate	4.4 (1.4–7.9)	6.8 (4.7–8.1)	7.9 (6.5–10.8)	4.2 (1.2–5.9)^*^
Isovalerate	8.5 (5.5–11)	11.7 (6.3–15)	13.2 (8–15.5)	6.7 (5.5–10.6)^*^

Data are expressed as median (IQR); values within same dietary pattern and row are significant at

* p < 0.05 and

† *p < 0.1*;

*Only bacteria that showed significant difference between the groups of dietary pattern are shown; VFA, volatile fatty acids*.

In DP1, children above the median had higher intakes of fruit, vegetables, legumes, nuts and seeds, refined carbohydrates and starchy vegetables, but lower intakes of sweets compared to children falling below the median. Additionally, children above the median in DP1 had higher intake of folate, vitamin E, vitamin A, and insoluble dietary fiber, but lower intakes of vitamin B12. Additionally, children above the median in DP1 had lower abundance of Enterobacteriaceae, *Lactococcus, Roseburia, Leuconostoc*, and *Ruminococcus* compared to children below the median. No significant differences in VFA concentrations at baseline between factor score categories in DP1 were observed. Lastly, children above the median in DP had lower total GI severity and constipation scores.

In DP2, children above the median had lower intakes of vegetables, legumes, nuts and starchy vegetables. Furthermore, children above the median in DP2 had higher intakes of vitamin B12 as well as total and refined grains, but lower intake of niacin and vitamin B6 compared to children below the median in DP2. Additionally, children above the median in DP2 had higher abundance Barnesiellaceae and *Alistipes* and lower abundance of Streptophyta. Higher levels of propionate, isobutyrate, valerate and isovalerate were observed in children above the median in DP2.

## Discussion

A GI microbial dysbiosis in children with ASD has been increasingly described and some bacterial taxa are suggested to influence symptoms associated with ASD (De Angelis et al., 2013; Son et al., 2015; Tomova et al., 2015). Even though diet represents a major environmental factor that influences GI microbiota composition and inadequate nutrient intake is often reported in children with ASD (Cermak et al., 2010), studies investigating the microbiota in children with ASD have not systematically investigated the diet-microbiota interaction in this population. Therefore, we collected information on dietary habits (food frequency questionnaire, 3-day food diary) as well as fecal samples from children with ASD and unaffected controls to investigate how nutrient intake and dietary patterns impact the GI microbiota composition. Additionally, specific nutrients or food groups were evaluated for their potential to influence the microbiota-brain connection in ASD. Differences in the microbiota composition and microbial metabolites between children with ASD and unaffected controls were described which are in accordance with previously published literature. Additionally, 2 bacterial taxa were identified which positively predict social deficit scores. Distinct microbiota profiles based on dietary habits that were related to GI severity symptoms were identified, but diet-induced microbial composition was not associated with social deficit scores. To the best of our knowledge, this is the first study reporting an association between dietary intake and microbiota composition in children with ASD.

There is now growing evidence for an association between individual bacteria and symptoms of ASD. For example, bacterial richness, lower Bacteroidetes-to-Firmicutes ratio and abundance of *Desulfovibrio* were related to ASD symptoms (Kang et al., 2013; Tomova et al., 2015). Here, using regression analysis, we identified that the abundance of Peptostreptococcaceae and *Faecalibacterium* were strong positive predictors of the social deficit score in children with ASD. Neither of these taxa have previously been associated with symptoms of ASD. A role of *Faecalibacterium* in health and disease has been suggested. Although *F. prausnitzii*, to date the only known species within the *Faecalibacterium* genus, is usually regarded as a beneficial bacterium due to its anti-inflammatory properties (Quévrain et al., 2016), improvements of GI barrier function (Carlsson et al., 2013) and support of mucosal immune homeostasis (Hornef and Pabst, 2016), other studies demonstrated that *Faecalibacterium* could be associated with some disease states (Swidsinski et al., 2008; Hansen et al., 2012). Peptostreptococcaceae, the second bacterium associated with social deficit symptoms in this cohort, is a family within the order of Clostridiales and encompasses species such as *C. difficile* and other pathogenic clostridia (Milani et al., 2016). Although some studies suggest that Peptostreptococcaceae could contribute to GI homeostasis (Fan et al., 2017), higher abundances in patients with IBD, Ulcerative Colitis, and colorectal cancer were reported (Chen et al., 2012; Lavelle et al., 2015). Furthermore, an overrepresentation of Peptostreptococcaceae in a mice model of colitis and association with intestinal mucosal ulceration suggests an influence of that family on an inflammatory status (Nagy-Szakal et al., 2013; Denis et al., 2016). Lastly, correlations between Peptostreptococcaceae and the right inferior segment of the circular sulcus in patients with IBS, a region for somatosensory and motor function, indicate the potential of this family to impact brain and behavior (Labus et al., 2017).

Of the studies analyzing the GI microbiota composition in children with ASD, only Son and colleagues (Son et al., 2015) collected dietary information and analyzed the macronutrient intake of study participants. However, the impact of dietary intake on the GI microbiota composition was not systematically investigated. Other studies only collected information on specialty diets (Horvath et al., 1999; Finegold et al., 2002; Parracho et al., 2005; Wang et al., 2011; Kang et al., 2013) or probiotic and supplement use (Adams et al., 2011; Wang et al., 2011; Kang et al., 2013). It is well accepted that the GI microbiota and bacterial metabolites are associated with nutrient intake and dietary patterns. Thus, we applied several different approaches to investigate the impact of eating habits and nutrient intake on the GI microbiota in children with ASD.

First, we investigated whether picky eating behavior, diet variety or repetitive eating patterns were associated with a distinct dietary intake and microbiota composition in children with ASD. Picky eating behaviors and repetitive eating pattern are commonly observed in children with ASD (Provost et al., 2010; Diolordi et al., 2014). In this study population, about half of the children with ASD were perceived by their parents as being picky eaters or having a repetitive eating pattern. Differences in nutrient intake and microbial profile were observed in children with ASD based on eating behaviors. Some bacteria that were associated with picky eating, low dietary variety or repetitive eating patterns have been previously described as being associated with host physiology. For example, abundance of Bacteroides and *Phascolarctobacterium* were lower in children with ASD and picky eating behavior. Higher abundance of Bacteroides has been shown to support the development of immune tolerance and avoidance of allergy and asthma (Wexler, 2007). Higher *Phascolarctobacterium* abundance can benefit the host by producing VFA (Louis et al., 2010) and has been correlated with positive mood (Li et al., 2016). Additionally, higher levels of Coriobacteriaceae, as observed in children with ASD and picky eating behavior and repetitive eating patterns, was associated with host inflammatory status (Qasem et al., 2017). Higher abundances of Actinobacteria as well as *Bifidobacterium*, which belongs to the phylum Actinobacteria, were detected in children with ASD who consumed few than 20 foods in their diet. Actinobacteria abundance was reported to be higher in children with Attention Deficit/Hyperactivity Disorder (Aarts et al., 2017) and was positively associated with brain structure and function in adults (Fernandez-Real et al., 2015). *Bifidobacterium* is regarded as a beneficial microbe due to its ability to improve epithelial barrier function, to reduce allergic symptoms, to prevent pathogen infections, and to produce bioactive metabolites, such as VFA, vitamins or polyunsaturated fatty acids, which contribute to intestinal function and immune modulation (Bottacini et al., 2014; Hidalgo-Cantabrana et al., 2017). However, higher abundance of *Bifidobacterium* could also indicate a less mature microbiota composition, since *Bifidobacterium* decreases with age (Ottman et al., 2012). Furthermore, the abundance of another potentially pathogenic bacteria, Clostridiales, was higher in children with ASD with less than 20 foods in their diet and repetitive eating patterns. The abundances of Clostridiaceae and *Clostridium* are often associated with ASD and species within the Clostridiaceae family could potentially affect ASD symptomology by producing entero- and neurotoxins (Finegold, 2008). Lastly, higher concentrations of isobutyrate and isovalerate in picky eaters could suggest microbial metabolic changes and increased dietary energy extraction form the microbiota in picky eaters among children with ASD. Whether isobutyrate is beneficial or harmful remains to be determined (Jost et al., 2014). In states of low butyrate concentrations, colonocytes can metabolize isobutyrate (Jaskiewicz et al., 1996) and the microbiota of patients with IBS produced 25% more branched chain fatty acids compared to healthy individuals (Van Nuenen et al., 2004).

Second, we performed simple correlation analysis to determine whether dietary intake correlates with individual bacterial taxa and VFA concentrations. In accordance with the commonly accepted knowledge that the GI microbiota and bacterial metabolites are associated with nutrient intake and dietary patterns, we found correlations between intake of specific nutrients and the abundance of microbiota and VFA concentrations in both children with ASD and unaffected controls. Some bacteria commonly affected by dietary intake include *Clostridium* and *F. prausnitzii*, as reported herein (Singh et al., 2017). Thereby, different food groups and different macronutrients can have distinct effects on the microbiota composition and microbial metabolites. Associations between macronutrient intake and bacterial abundance previously reported are in line with correlations observed herein. For example, dairy intake was negatively associated with species richness and diversity, whereas vegetable intake increased *Lachnospira* abundance and fruit intake decreased Firmicutes:Bacteroidetes ratio and *Ruminococcus gnavus* abundance (Smith-Brown et al., 2016). Additionally, insoluble fiber negatively correlated with Clostridiales abundance. Interestingly, in children with ASD abundance of *Faecalibacterium* positively correlated with unhealthy food group (fried food), but negatively correlated with a beneficial food (fruit). Previous research has shown that a healthy dietary pattern, the Mediterranean diet, increased the abundance of *F. prausnitzii* (Haro et al., 2016). These results provide first evidence that nutrient and food group intake influence the microbiota composition and VFA concentration in children with ASD and suggest that dietary intake should be considered when analyzing microbial composition in this population.

Thirdly, we described 2 dietary patterns in children with ASD that are linked to a distinct microbiota composition. Microbiota composition can be clustered based on dietary habits (Muegge et al., 2011) and healthier long-term dietary patterns, i.e., increased consumption of fruits, vegetables and whole grains and lower intake of processed foods, simple carbohydrates and fried foods has been associated with a microbial profile that could potentially protect against diseases (Albenberg and Wu, 2014). Defining long-term dietary patterns that are associated with a beneficial microbial profile for symptoms or associated symptoms of ASD could provide new guidelines for future intervention strategies. Herein, an eating pattern characterized by higher intake of healthy foods such as fruit, vegetables and legumes, nuts and seeds was associated with a bacterial profile that could potentially be related to some aspects of GI health in accordance with the observation of lower GI scores in children harboring this microbial profile. For example, higher abundance of Enterobacteriaceae was proposed as marker of microbiota dysbiosis and epithelial dysfunction (Shin et al., 2015) and increased abundance of members of the Enterobacteriaceae family is often observed in patients with Crohn's disease and ulcerative colitis (Frank et al., 2007). Furthermore, the abundance of Enterobacteriaceae in mice fed a high-fat diet was correlated with an increase in endotoxin concentrations (Kim et al., 2012). In children with ASD, Enterobacteriaceae levels were highest compared to unaffected controls and children with Pervasive Developmental Disorder not otherwise specified (De Angelis et al., 2013). Additionally, some reports of *Leuconostoc* bacteremia (Casanova-Román et al., 2003; Ishiyama et al., 2011) suggest that some species within this genus may be opportunistic pathogens. *Roseburia*, known for its health benefits associated with butyrate production (Tamanai-Shacoori et al., 2017), was shown to be positively correlated with BMI and systemic inflammation in obese subjects (Verdam et al., 2013) and could be involved in the development of insulin resistance in mice fed a high-fat high-sugar diet (Org et al., 2015). These observations suggest that the physiological effect of *Roseburia* and other beneficial microbes depends on substrate availability, presence of other microbes and physiological condition of the host and, thus, should be interpreted within this broader context.

For DP2, children that were above the median had lower intakes of vegetables, legumes, nuts and starchy vegetables and had higher intakes of total and refined grains compared to children below the median in DP2. This eating pattern richer in processed foods was associated with a microbiota composition that harbored more less beneficial microbes. Barnesiellaceae, which was increased in children above the median in DP2, has been associated with sedentary lifestyles and predicted by the percentage of body fat (Bressa et al., 2017). Furthermore, the abundance of *Alistipes* might be associated with increased abdominal pain, GI inflammation, systemic infections and major depressive disorder (Boente et al., 2010; Saulnier et al., 2011; Naseribafrouei et al., 2014; Jiang et al., 2015). Interestingly, children above the median had higher levels of propionate, and the branched chain fatty acids isobutyrate, valerate and isovalerate. Increased VFAs have been reported in children with ASD (Wang et al., 2012) and some studies suggest that VFAs could play a role in ASD pathophysiology (MacFabe et al., 2011; Thomas et al., 2012; Foley et al., 2015). Even though children above the median in DP2 displayed an unhealthier eating pattern and a less beneficial microbiota, no differences were observed in measures of host physiology, such as GI symptoms or social deficit scores.

Due to the profound effect of diet on the GI microbiota composition and the newly acquired knowledge about the gut-brain-connection, the GI microbiota was proposed to be key mediator in the diet-brain health connection potentially through interaction of the GI microbiota and its metabolic products with enteric neurons (Furness et al., 1999; Hanstock et al., 2004; Dawson et al., 2016). In ASD, dietary interventions to manipulate the GI microbiota and ameliorate some symptoms of ASD could be a promising therapeutic avenue (Newell et al., 2016). Contrary to our hypothesis, no associations between diet-induced microbial profile and social deficit scores were observed. Even though we did not find associations between a core symptom of ASD, social deficit scores, and diet-induced microbial profiles, scores for some GI symptoms were associated with dietary patterns and eating behavior, suggesting that some eating behaviors could potentially affect GI health through the microbiota in the ASD population. The commonly observed GI symptoms in the ASD population have been suggested to contribute to behavioral problems and to correlate with symptom severity (Horvath et al., 1999; Adams et al., 2011; Tomova et al., 2015); thus, promoting an eating pattern that promotes a microbial profile associated with fewer GI symptoms in the subpopulation of children with ASD and co-morbid GI problems might be a potential therapeutic avenue to alleviate some associated symptoms of ASD. However, future studies using more comprehensive GI assessment tools are needed to confirm the results reported herein.

The lack of association between diet-induced microbial profiles and social deficit scores could partly be attributable to the limited sample size. Likewise, it could be possible that repetitive or restrictive behaviors or other externalizing behaviors associated with ASD could be more significantly impacted by the microbiota composition. Previous studies demonstrated that problematic eating behaviors could be a manifestation of repetitive behaviors, ritualizing or externalizing behaviors of ASD instead of social communication deficit (Johnson et al., 2014). Furthermore, one study investigating the relationship between individual bacterial taxa and symptoms of ASD found that *Desulfovibrio* was strongly correlated with the restricted/repetitive behavior subscale score (Tomova et al., 2015). Due to the age of the participants in this study, only social deficits were measured as the only validated parent questionnaire that is available for children under the age of 4 is the PDDBI-SV. Thus, larger studies in the future should include measurements of all symptoms of ASD in order to provide more evidence for a relationship between diet-microbiota-symptoms in children with ASD.

Despite these limitations, using various approaches new evidence for a relationship between diet and microbiota in ASD is reported, which have not previously been described in the ASD population. This approach could provide valuable insight for future studies aimed at deciphering the relationship between microbiota and ASD symptoms. Additionally, intervention trials in children with ASD that evaluate the baseline microbiota and investigate relationships between changes in the microbiota and ASD symptoms are needed to elucidate whether dietary intervention can alleviate symptoms of ASD in a subgroup of individuals with a specific microbial profile.

## Ethics statement

This study was carried out in accordance with the recommendations of protocol for human subject research of the Institutional Review Board of the University of Illinois at Urbana-Champaign. The protocol was approved by the Institutional Review Board of the University of Illinois at Urbana-Champaign. All subjects gave written informed consent in accordance with the Declaration of Helsinki.

## Authors contributions

KB contributed to the design of the study, and was responsible the acquisition, analysis and interpretation of data for the work. She prepared the first draft and approved the final version of the manuscript. SD contributed to the design of the study, interpretation of data for the work and reviewed and approved the final version of the manuscript.

### Conflict of interest statement

The authors declare that the research was conducted in the absence of any commercial or financial relationships that could be construed as a potential conflict of interest.
