# Supplementary material for: Diet Can Impact Microbiota Composition in Children With Autism Spectrum Disorder
Source: Front Neurosci. 2018 Jul 31;12:515. doi: 10.3389/fnins.2018.00515 (PMC6079226; doi:10.3389/fnins.2018.00515)
Supplement: Supplementary file 1 [file Data_Sheet_1.pdf]

Supplementary Material

Diet Can Impact Microbiota Composition in Children With Autism Spectrum Disorder

Kirsten Berding*, Sharon Donovan

*** Correspondence:** Corresponding Author: Sharon Donovan, sdonovan@illinois.edu

1 Supplementary Figures and Tables

1.1 Supplementary Tables

Supplemental Table 1. Primer for Real-time quantitative analysis of microbial populations.

Target Group	Primer	Sequence (5’ – 3’)	Annealing Temperature (°C)	Reference
Total Bacteria	Uni331F<br>Uin797R	TCCTACGGGAGGCAGCAGT<br>GGACTACCAGGGTATCTATCCTGTT	60	Nadkarni et al, 2002
Lactobacillus spp.	LacF<br>LacR	AGCAGTAGGGAATCTTCCA<br>CACCGCTACACATGGAG	58	Kok et al, 1996
Bifidobacterium spp.	Bif164F<br>Bif662R	GGGTGGTAATGCCGGATG<br>CCACCGTTACACCGGGAA	60	Kok et al, 1996
Prevotella spp.	g_prevF<br>g_prevR	GGTTCTGAGAGGAAGGTCCCC<br>TCCTGCACGCTACTTGGCTG	55	Rinttila et al., 2004
Clostridium perfringens	s-Clper-F<br>ClPER-R	GGGGGTTTCAACACCTCC<br>GCAAGGGATGTCAAGTGT	60	Matsuda et al., 2009
Clostridium difficile	Cdiff_F<br>Cdiff_R	TTGAGCGATTTACTTCGGTAAAGA<br>CCATCCTGTACTGGCTCACCT	58	Rinttila T, 2004
mmdA	mmdAF<br>mmdAR	AATGACTCGGGIGGIGCIMGNATHCARGA<br>GATTGTTACYTTIGGIACNGTNGCYTC	56	Reichardt et al, 2014
BCoAT	BCoATscrF<br>BCoATscrR	GCIGAICATTTCACITGGAAYWSITGGCAYATG<br>CCTGCCTTTGCAATRTCIACRAANGC	53	Louis and Flint 2007

Supplemental Table 2.

(A) Relative abundances of bacterial taxa detected in feces of children in the ASD and CONT groups.

**Phyla**	**ASD (n=7)**	**CONT (n=32)**
Euryarchaeota	0.51 ± 2.03	0.11 ± 0.63
Actinobacteria	5.98 ± 5.96	3.92 ± 5.85
Bacteroidetes	39.88 ± 20.75†	49.5 ± 19.02
Cyanobacteria	0.003 ± 0.007	0.01 ± 0.02
Firmicutes	50.53 ± 18.16*	40.42 ± 14.47
Fusobacteria	0.0002 ± 0.0008	0.0003 ± 0.001
Proteobacteria	0.85 ± 1.03	0.99 ± 1.23
Tenericutes	0.0002 ± 0.0004	0.0002 ± 0.0003
Verrucomicrobia	2.26 ± 4.18	5.39 ± 10.3

**Order**	**ASD (n=26)**	**CONT (n=32)**
Bacteroidales	0.02 ± 0.06	0.00 ± 0.00
Streptophyta	0.00 ± 0.01†	0.01 ± 0.02
Clostridiales	3.98 ± 2.73†	2.47 ± 1.3
RF32	0.02 ± 0.05	0.01 ± 0.05
RF39	0.00 ± 0.00	0.00 ± 0.00

**Family**	**ASD (n=26)**	**CONT (n=32)**
Coriobacteriaceae	0.29 ± 0.54*	0.05 ± 0.1
RF16	0.01 ± 0.02	0.00 ± 0.01
Rikenellaceae	2.95 ± 4.67*	3.83 ± 3.27
S247	0.00 ± 0.00	0.03 ± 0.12
Barnesiellaceae	0.88 ± 1.62	1.57 ± 3.58
Christensenellaceae	0.13 ± 0.3	0.19 ± 0.93
Clostridiaceae	1.65 ± 2.96†	0.32 ± 0.36
EtOH8	0.01 ± 0.03	0.00 ± 0.01
Lachnospiraceae	8.39 ± 4.62	6.71 ± 3.48
Peptostreptococcaceae	0.12 ± 0.21*	0.03 ± 0.05
Mogibacteriaceae	0.03 ± 0.06	0.03 ± 0.04
Erysipelotrichaceae	0.86 ± 1.11	0.68 ± 0.89
Enterobacteriaceae	0.26 ± 0.49	0.10 ± 0.25

**Genera**	**ASD (n=26)**	**CONT (n=32)**
Archea – Euryarchaeota		
Methanobrevibacter	0.51 ± 2.03	0.12 ± 0.63
Actinobacteria		
Bifidobacterium	3.33 ± 3.89	3.20 ± 4.61
Adlercreutzia	0.16 ± 0.24	0.14 ± 0.27
Collinsella	1.93 ± 3.6	0.39 ± 1.6
Eggerthella	0.15 ± 0.27	0.08 ± 0.13
Slackia	0.13 ± 0.39	0.06 ± 0.31

Bacteroidetes		
Bacteroides	30.50 ± 19.44[b]	38.14 ± 19.23
Parabacteroides	2.05 ± 2.85	2.09 ± 1.86
Prevotella	3.16 ± 8.81	2.98 ± 7.97
Alistipes	0.05 ± 0.16	0.02 ± 0.04
Butyricimonas	0.01 ± 0.04*	0.03 ± 0.11
Odoribacter	0.11 ± 0.2	0.29 ± 0.52
Paraprevotella	0.03 ± 0.11	0.15 ± 0.58
Prevotella	0.11 ± 0.4	0.01 ± 0.05
Firmicutes		
Staphylococcus	0.02 ± 0.06	0.01 ± 0.02
Enterococcus	0.16 ± 0.52	0.00 ± 0.02
Lactobacillus	0.09 ± 0.27	0.00 ± 0.00
Leuconostoc	0.03 ± 0.15	0.00 ± 0.00
Lactococcus	0.09 ± 0.26	0.01 ± 0.00
Streptococcus	0.51 ± 0.87	0.29 ± 0.43
Turicibacter	0.34 ± 0.98	0.14 ± 0.46
02d06	0.01 ± 0.01	0.00 ± 0.00
Clostridaceae_Clostridium	0.86 ± 1.87*	0.20 ± 0.41
SMB53	2.64 ± 3.59†	1.24 ± 1.59
Sarcina	0.05 ± 0.13	0.00 ± 0.00
Eubacterium	0.01 ± 0.03	0.00 ± 0.01
Anaerostipes	0.10 ± 0.16	0.12 ± 0.19
Blautia	1.84 ± 1.41*	1.18 ± 0.93
Butyrivibrio	0.01 ± 0.02*	0.05 ± 0.05
Coprococcus	3.88 ± 2.32	2.97 ± 1.9
Dorea	1.13 ± 0.98	0.84 ± 0.57
Lachnospira	0.47 ± 0.9	0.49 ± 0.57
Roseburia	1.02 ± 1.94*	0.43 ± 0.55
Peptococcus	0.06 ± 0.22	0.00 ± 0.01
Anaerotruncus	0.02 ± 0.04	0.01 ± 0.01
Peptostreptococcaceae_Clostridium	0.007 ± 0.03	0.002 ± 0.003
Faecalibacterium	8.02 ± 7.03*	11.13 ± 6.46
Oscillospira	0.49 ± 0.34	0.47 ± 0.26
Ruminococcus	2.83 ± 2.9	2.55 ± 2.97
Acidaminococcus	0.02 ± 0.07	0.00 ± 0.02
Dialister	0.61 ± 1.04*	1.23 ± 1.57
Megamonas	0.57 ± 2.87	0.15 ± 0.59
Megasphaera	0.18 ± 0.92	0.00 ± 0.00
Phascolarctobacterium	0.31 ± 0.92	0.34 ± 0.94
Succiniclasticum	0.00 ± 0.00	0.04 ± 0.24
Veillonella	0.10 ± 0.32	0.04 ± 0.07
Catenibacterium	0.04 ± 0.14	0.00 ± 0.00
Coprobacillus	0.01 ± 0.03	0.01 ± 0.02
Holdemania	0.01 ± 0.01	0.01 ± 0.01
Fusobacteria		
Fusobacterium	0.00 ± 0.00	0.00 ± 0.00

Proteobacteria		
Sutterella	0.28 ± 0.37	0.61 ± 0.76
Bilophila	0.03 ± 0.06*	0.04 ± 0.06
Campylobacter	0.05 ± 0.23	0.00 ± 0.00
Haemophilus	0.22 ± 0.68	0.22 ± 0.87
Verrucomicrobia		
Akkermansia	2.26 ± 4.18	5.39 ± 10.3

(B) Bacterial Densities in feces of children in the CONT and ASD groups.

	**ASD**	**CONT**
Log_{10} gene copy numbers/g		
Total Bacteria	12.34 ± 0.83	12.71 ± 0.67
Lactobacillus	7.39 ± 1.14	7.59 ± 0.93
Bifidobacterium	9.15 ± 1.04*	9.82 ± 0.56
Prevotella	10.45 ± 0.94	11.03 ± 0.78
Clostridium perfringens	6.62 ± 0.58*	7.38 ± 1.14

Data expressed as mean ± SD; data was analyzed using proc glimmix (non-normal data); within same segment and row, different from CONT at *p≤0.05 and †≤0.1; age, gender, height, weight, BMI and season were included as covariates; ASD=Autism Spectrum Disorder group; CONT=unaffected control group

Supplemental Table 3. Comparison of Nutrient Intake and Food Groups Consumed by Children in the ASD and CONT groups.

(A) Nutrient intake

•

Variable	ASD (n=26)	CONT (n=32)
**Macronutrients**		
Energy	1371 ± 311	1489 ± 306
Total Fat	51 ± 16	56 ± 17
Omega-6 Fatty Acids	11 ± 4.4	11 ± 4.7
Omega-3 Fatty Acids	1.1 ± 0.5	1.2 ± 0.6
SFA	18 ± 7	14 ± 4
MUFA	18 ± 5	20 ± 7
PUFA	12 ± 5	9 ± 2
Total Carbohydrate	184 ± 44	147 ± 67
Total Sugars	79 ± 32	88 ± 28
Added Sugars	44 ± 24	46 ± 24
Total Grains	5.6 ± 2.1	6 ± 1.9
Whole Grains	1.2 ± 1.6	1.3 ± 1.1
Refined Grains	4.4 ± 2	4.7 ± 2
Total Protein	49 ± 16	55 ± 18
**Dietary Fiber**		
Total Dietary Fiber	12.3 ± 4.9	14.6 ± 5.6
Soluble Dietary Fiber	4 ± 1.6	3.1 ± 1.6
Insoluble Dietary Fiber	8.2 ± 3.6†	10.3 ± 4.2
Pectin	1.5 ± 0.8†	0.6 ± 0.6
**Vitamins**		
Vitamin A	523 ± 400	405 ± 177
Vitamin D	4.0 ± 3.1	4.8 ± 2.1
Vitamin E	6.4 ± 2.5	5.9 ± 1.8
Vitamin K	43.7 ± 32	58.8 ± 53.1
Vitamin C	52.4 ± 43.6†	80.5 ± 51.1
Thiamin	1.2 ± 0.4	1.3 ± 0.4
Riboflavin	1.5 ± 0.5	1.6 ± 0.4
Niacin	15.3 ± 5.1	15.9 ± 6.1
Pantothenic Acid	3.3 ± 1.2	3.6 ± 1.1
Vitamin B_6	1.3 ± 0.5	1.3 ± 0.6
Folate	304 ± 231	344 ± 195
Vitamin B_{12}	3.5 ± 2.7	3.5 ± 1.4
**Minerals**		
Calcium	736 ± 318	798 ± 276
Phosphorus	875 ± 310	956 ± 235
Magnesium	178.6 ± 65	202.9 ± 61.4
Iron	12.3 ± 7.8	11.9 ± 6.5
Zinc	6.8 ± 3.4	8 ± 3.1
Copper	0.8 ± 0.6	0.9 ± 0.3
Selenium	67.5 ± 21.1	78.7 ± 25.9

Sodium	2029 ± 669	2092 ± 642
Potassium	1628 ± 628	1821.8 ± 585
Manganese	2.2 ± 0.9	2.7 ± 1.3
Choline	186 ± 71	216 ± 90

(B) Food Groups

**Food Group**	**ASD (n=26)**	**CONT (n=32)**
Fruit	2.27 ± 1.31	2.4 ± 1.04
Vegetables	1.16 ± 0.82	1.7 ± 0.85
Legumes	0.38 ± 0.49	0.40 ± 0.31
Starchy Foods	0.29 ± 0.33	0.35 ± 0.39
Starchy Vegetables	0.73 ± 0.64	0.53 ± 0.35
Juice	0.66 ± 0.61	0.39 ± 0.35
Sweetened Beverages	0.27 ± 0.31	0.26 ± 0.29
Grains	0.86 ± 0.94	0.8 ± 0.70
Refined Carbohydrates	1.13 ± 0.72	0.99 ± 0.41
Fried Foods	0.33 ± 0.31	0.25 ± 0.19
Protein	1.39 ± 0.89	1.2 ± 0.63
Dairy	3.26 ± 1.68*	4.5 ± 1.94
Snack	1.17 ± 0.71†	0.8 ± 0.51
Sweets	2.17 ± 0.57†	1.7 ± 0.8
Kid's Meal	0.87 ± 0.57	1 ± 0.52
Fish	0.12 ± 0.12	0.16 ± 0.14
Condiments	0.40 ± 0.39	0.38 ± 0.29

Food groups consumed were derived from YAQ; nutrient intake derived from three day food record; Differences between groups examined with proc glimmix (non-normal data) and proc mixed (normal data) with age, gender, height, weight, BMI and season were included as co-variates; within same segment and row, different from CONT at *p≤0.05 and †≤0.1; ASD, Autism Spectrum Disorder group; CONT, unaffected control group

Supplemental Table 4. Correlations between dietary intake and food groups with bacterial phyla/genera in ASD (a) and CONT (b).
